# Investigation about Personality Characteristics and Acute Symptom Response Predict Chronic Symptoms after traumatic brain injuries in young male athletes

**DOI:** 10.1192/j.eurpsy.2026.11903

**Published:** 2026-07-31

**Authors:** N. Syrmos

**Affiliations:** Aristotle University, Thessaloniki, Greece

## Abstract

**Introduction:**

Aim- Evaluation of the diverse personality and psychiatric symptom dimensions predict in traumatic brain injury recovery in young male athletes.

**Objectives:**

Material –Methods**-** This retrospective study involved psychological assessments of young male patients with traumatic brain injuries (n = 40, median = 2 days post-injury, range = 0-12 days, 15-35 years old, median age 25) .Chronic symptoms were evaluated at 6 months and in 1 year period after the traumatic events using the Sport Concussion Assessment Tool (SCAT) symptom checklist.

**Methods:**

Results:**-** Radiological investigation with MRI studies, neurological and psychiatric evaluation in all 40 cases-100%. In these traumatic events, trait psychoticism directly predicted chronic traumatic symptoms and was the strongest personality predictor overall (30 cases, 75%).

**Results:**

Also internalizing personality dimension emphasizing negative affect/emotionality and detachment predicted chronic traumatic symptoms indirectly through enhancement of acute somatic complaints (20 cases, 50%). %. Good rehabilitation in 30 cases, 75% and moderate in 10 cases, 25%.

**Conclusions:**

Conclusions- Multiple combined approaches of various medical disciplines is more than necessary in order to achieve optimal results.

**Disclosure of Interest:**

None Declared

